# Cardiovascular Risk Factors as Independent Predictors of Diabetic Retinopathy in Type II Diabetes Mellitus: The Development of a Predictive Model

**DOI:** 10.3390/medicina60101617

**Published:** 2024-10-02

**Authors:** Cristian Dan Roşu, Melania Lavinia Bratu, Emil Robert Stoicescu, Roxana Iacob, Ovidiu Alin Hațegan, Laura Andreea Ghenciu, Sorin Lucian Bolintineanu

**Affiliations:** 1 1st Surgery Clinic ‘Victor Babes’, University of Medicine and Pharmacy Timisoara, Eftimie Murgu Square No. 2, 300041 Timisoara, Romania; rosu.cristian@umft.ro; 2 Center for Neuropsychology and Behavioral Medicine, Discipline of Psychology, Faculty of General Medicine, ‘Victor Babes’ University of Medicine and Pharmacy Timisoara, Eftimie Murgu Square No. 2, 300041 Timisoara, Romania; 3 Center for Cognitive Research in Neuropsychiatric Pathology, Department of Neurosciences, ‘Victor Babes’ University of Medicine and Pharmacy Timisoara, Eftimie Murgu Square No. 2, 300041 Timisoara, Romania; 4 Department of Radiology and Medical Imaging, ‘Victor Babes’ University of Medicine and Pharmacy Timisoara, Eftimie Murgu Square No. 2, 300041 Timisoara, Romania; stoicescu.emil@umft.ro; 5 Research Center for Pharmaco-Toxicological Evaluations, ‘Victor Babes’ University of Medicine and Pharmacy Timisoara, Eftimie Murgu Square No. 2, 300041 Timisoara, Romania; 6 Field of Applied Engineering Sciences, Specialization Statistical Methods and Techniques in Health and Clinical Research, Faculty of Mechanics, ‘Politehnica’ University Timisoara, Mihai Viteazul Boulevard No. 1, 300222 Timisoara, Romania; roxana.iacob@umft.ro; 7 Department of Anatomy and Embriology, ‘Victor Babes’ University of Medicine and Pharmacy Timisoara, 300041 Timisoara, Romania; s.bolintineanu@umft.ro; 8 Discipline of Anatomy and Embriology, Medicine Faculty, ‘Vasile Goldis’ Western University of Arad, Revolution Boulevard 94, 310025 Arad, Romania; hategan.ovidiu@uvvg.ro; 9 Department of Functional Sciences, ‘Victor Babes’ University of Medicine and Pharmacy Timisoara, Eftimie Murgu Square No. 2, 300041 Timisoara, Romania; bolintineanu.laura@umft.ro; 10 Center for Translational Research and Systems Medicine, ‘Victor Babes’ University of Medicine and Pharmacy Timisoara, Eftimie Murgu Square No. 2, 300041 Timisoara, Romania

**Keywords:** diabetic retinopathy, type 2 diabetes mellitus, cardiovascular risk factors, predictive model, blood pressure, BMI, lipid profile

## Abstract

*Background*: Diabetic retinopathy (DR) is a leading cause of blindness in patients with type 2 diabetes mellitus (T2DM). Cardiovascular risk factors, such as hypertension, obesity, and dyslipidemia, may play a crucial role in the development and progression of DR, though the evidence remains mixed. This study aimed to assess cardiovascular risk factors as independent predictors of DR and to develop a predictive model for DR progression in T2DM patients. *Methods*: A retrospective cross-sectional study was conducted on 377 patients with T2DM who underwent a comprehensive eye exam. Clinical data, including blood pressure, lipid profile, BMI, and smoking status, were collected. DR staging was determined through fundus photography and classified as No DR, Non-Proliferative DR (NPDR), and Mild, Moderate, Severe, or Proliferative DR (PDR). A Multivariate Logistic Regression was used to evaluate the association between cardiovascular risk factors and DR presence. Several machine learning models, including Random Forest, XGBoost, and Support Vector Machines, were applied to assess the predictive value of cardiovascular risk factors and identify key predictors. Model performance was evaluated using accuracy, precision, recall, and ROC-AUC. *Results*: The prevalence of DR in the cohort was 41.6%, with 34.5% having NPDR and 7.1% having PDR. A multivariate analysis identified systolic blood pressure (SBP), LDL cholesterol, and body mass index (BMI) as independent predictors of DR progression (*p* < 0.05). The Random Forest model showed a moderate predictive ability, with an AUC of 0.62 for distinguishing between the presence and absence of DR XGBoost showing a better performance, featuring a ROC-AUC of 0.68, while SBP, HDL cholesterol, and BMI were consistently identified as the most important predictors across models. After tuning, the XGBoost model showed a notable improvement, with an ROC-AUC of 0.72. *Conclusions*: Cardiovascular risk factors, particularly BP and BMI, play a significant role in the progression of DR in patients with T2DM. The predictive models, especially XGBoost, showed moderate accuracy in identifying DR stages, suggesting that integrating these risk factors into clinical practice may improve early detection and intervention strategies for DR.

## 1. Introduction

Diabetes mellitus (DM) is a significant global health issue, affecting 422 million people worldwide, with most residing in low- and middle-income nations. DM is directly responsible for 1.6 million deaths annually. One of its major complications is diabetic retinopathy (DR), the most common cause of vision loss in people of working age [1]. DR has two stages: Non-Proliferative (NPDR) and Proliferative (PDR), both linked to hyperglycemia-induced retinal microvascular injury. Several metabolic pathways, including the polyol pathway, the accumulation of advanced glycation end products (AGEs), the protein kinase C (PKC) pathway, and the hexosamine pathway, are involved in this injury [2].

Cardiovascular risk factors such as hypertension play a crucial role in the development and progression of DR [3]. Hypertension increases retinal blood flow, exacerbating endothelial dysfunction and accelerating capillary damage in the retinal microvasculature. Elevated systolic and diastolic blood pressures are strongly correlated with the incidence and progression of DR, particularly PDR and diabetic macular edema (DME), independent of glycemic control [4,5].

Dyslipidemia, characterized by elevated levels of low-density lipoprotein cholesterol (LDL-C) and triglycerides, contributes to DR through mechanisms such as oxidative stress and endothelial dysfunction [6]. High levels of oxidized LDL (oxLDL) are cytotoxic to retinal endothelial cells, interacting with receptors like LOX-1 and triggering apoptosis. This process compromises the blood–retinal barrier, leading to increased vascular permeability, plasma leakage, and retinal edema [6,7]. Obesity, linked to DR through insulin resistance, systemic inflammation, and endothelial dysfunction, exacerbates the hyperglycemic environment and accelerates microvascular complications. Dysregulated adipokines and impaired nitric oxide (NO) production contribute to vascular permeability and neovascularization, worsening both NPDR and PDR [8,9]. Smoking is also an independent risk factor for DR, primarily due to its contribution to vascular inflammation, oxidative stress, and impaired endothelial function [10]. Nicotine reduces the bioavailability of NO, increasing vascular resistance and blood pressure in the retinal microvasculature, leading to capillary damage and retinal ischemia [11]. Smoking also increases the production of reactive oxygen species (ROS), amplifying oxidative stress and triggering inflammatory pathways [10]. It accelerates retinal microvascular damage and reduces the response to treatments like laser photocoagulation and anti-VEGF therapy [12].

The independent role of these cardiovascular risk factors in DR is further supported by large-scale epidemiological studies, which have consistently shown that tight control of blood pressure and lipid levels, alongside glycemic control, significantly reduces the risk of DR progression. For instance, the UK Prospective Diabetes Study (UKPDS) [13] and the Action to Control Cardiovascular Risk in Diabetes (ACCORD) trial [14] have provided evidence that multifactorial risk reduction strategies are essential in preventing vision loss due to DR.

Predictive models are crucial in healthcare, especially for managing chronic conditions like DR, where early detection can reduce the risk of severe complications. By integrating clinical variables such as BMI, blood pressure, and lipid levels, these models can identify individuals at higher risk of developing DR before symptoms appear [15,16]. This predictive capability allows healthcare providers to take proactive measures, such as adjusting treatment plans or recommending lifestyle changes. These models are also vital for efficient allocation of healthcare resources. They help prioritize high-risk patients for regular screening and early interventions, preventing progression to more severe stages that could lead to vision loss. Various ocular diseases, whether genetic [17], metabolic [2], or vascular [18], contribute to significant visual dysfunction. Predictive models offer insights that go beyond traditional clinical assessments by leveraging advanced algorithms and patient data. As the prevalence of diabetes rises globally [19], predictive models play an increasingly important role in identifying at-risk populations in regard to DR. This strategy is key in preventing blindness and reducing the burden of diabetic complications on healthcare systems [15].

The aim of this study is to evaluate the role of cardiovascular risk factors as independent predictors of DR in patients with T2DM. Furthermore, we seek to develop a predictive model that can effectively classify the presence and severity of DR based on these risk factors, providing insights into early detection and intervention strategies to prevent disease progression and vision loss.

## 2. Materials and Methods

### 2.1. Study Design

This study was a retrospective cross-sectional study designed to investigate the role of cardiovascular risk factors as independent predictors of DR in patients with T2DM. The study also aimed to develop a predictive model for DR progression, including stages of DR (Mild, Moderate, Severe, Proliferative). This study follows the TRIPOD (Transparent Reporting of a Multivariable Prediction Model for Individual Prognosis Or Diagnosis) guidelines to ensure transparent and comprehensive reporting of the predictive models. The study design, patient inclusion criteria, variable selection, model development, and performance evaluation metrics, such as accuracy, precision, recall, and ROC-AUC, are all presented in accordance with TRIPOD standards [20].

### 2.2. Patient Population

A total of 377 patients with a confirmed diagnosis of T2DM were included in the study. The data were collected from patients attending the outpatient ophthalmology clinic at the Timis County Emergency Clinical Hospital. Eligible patients were aged 18 years or older, had a diagnosis of diabetes for at least 1 year, and had undergone a comprehensive eye exam to assess the presence and stage of diabetic retinopathy. Exclusion criteria included patients with Type 1 diabetes, those who had undergone prior ocular surgery or retinal laser treatment, and patients with other retinal diseases that could interfere with the diagnosis of DR. The sample size was determined based on the number of available patients meeting the inclusion criteria. Considering the complexity of the model and aiming for at least 10 events per predictor variable, a minimum of 300 participants was required for adequate model development and validation.

### 2.3. Data Collection

The following clinical and demographic data (predictors) were collected: demographics (age, gender), body mass index (BMI); cardiovascular risk factors, including blood pressure (systolic and diastolic), lipid profile (LDL cholesterol, HDL cholesterol, triglycerides, total cholesterol), and smoking status; and diabetic retinopathy status, including the presence and stage of DR, classified as No DR, NPDR (which was also subclassified as Mild, Moderate, or Severe), and PDR based on fundus examination and retinal imaging (Table 1). The classification process was conducted by trained ophthalmologists who used a Haag-Streit biomicroscope (model BM 900, Haag-Streit AG, Berne, Switzerland, 2015) and Volk lens (Volk Optical, Inc., Mentor, OH, USA) for slit-lamp biomicroscopy, along with a Topcon non-mydriatic fundus camera (Topcon Medical Systems, Inc., Tokyo, Japan, 2017) for retinal imaging. The grading followed standardized criteria similar to the Early Treatment Diabetic Retinopathy Study (ETDRS) guidelines. Each patient underwent a detailed fundus examination, and the severity of the retinal changes was documented to ensure consistent and standardized classifications. Predictors were selected based on established associations with DR in prior research, and all data were measured using standard clinical protocols.

### 2.4. Handling of Missing Data

Missing data in this study were addressed using multiple imputation. The imputed datasets were analyzed and the results were combined to account for the variability introduced by the imputation process. This approach ensured that all cases were retained in the analysis, minimizing potential bias while maintaining the integrity of the dataset.

### 2.5. Statistical Analysis

Descriptive statistics were calculated for all variables. Categorical variables were presented as frequencies and percentages, while continuous variables were reported as means (standard deviations). A univariate analysis was performed to assess the association between cardiovascular risk factors and DR presence and progression. Chi-Square tests were used for categorical variables and t-tests were used for continuous variables.

A multivariate Logistic Regression was conducted to evaluate the independent effect of cardiovascular risk factors (BMI, blood pressure, lipid profile) on DR presence and stage, adjusting for potential confounders such as age and gender. The model also included interaction terms to explore potential synergies between risk factors. Machine learning models, such as Random Forest, XGBoost, Support Vector Machines (SVMs), and Logistic Regression were all applied to compare predictive performance. All models were validated using 5-fold cross-validation to ensure generalizability. Performance metrics for all models included accuracy, precision, recall, and ROC-AUC. Feature importance in predicting DR was assessed using a Random Forest model, which identified key predictors by assessing how much each variable reduced the error in the model. The models were trained using 70% of the dataset, and 30% was used for testing and validation. The model’s performance was evaluated using accuracy, precision, recall, and the confusion matrix. Following the initial evaluation of our models, we proceeded with hyperparameter tuning to further improve performance. Using a grid search and cross-validation approach, we optimized key hyperparameters for the XGBoost, Random Forest, and SVM models. For XGBoost, parameters such as the learning rate, maximum tree depth, and number of estimators were fine-tuned. For Random Forest, we adjusted the number of trees and the maximum depth, while, for SVMs, we tuned the regularization parameter and the kernel function. All statistical analyses were performed using Python and SPSS software (version 29), and a *p*-value of <0.05 was considered statistically significant.

### 2.6. Ethics Approval

This study was approved by the Institutional Review Board of Timis County Emergency Clinical Hospital and informed consent was obtained from all participants. The study adhered to the principles of the Declaration of Helsinki.

## 3. Results

The study population comprised 377 type II diabetic patients (Table 2) with a mean age of 55.15 years and a median age of 55 years. The most frequent age mode was 66 years, with ages spanning from 18 to 86 years. In terms of gender, 47.2% of the patients were male (N = 178) and 52.8% were female (N = 199), indicating a slight predominance of females in the sample. The average age for males was 53.99 years, while, for females, it was 56.16 years. Both genders had the same most frequent age of 66 years, and the age range for both males and females was 18 to 86 years. The analysis revealed that there is no statistically significant difference in the ages between male and female patients, as indicated by an independent t-test (t = −0.24, *p* = 0.81).

In this study, the 377 patients were classified into five categories depending on their retinal fundus changes. The majority of patients, 220 individuals (58.4%), showed No DR. A significant portion, 60 patients (15.9%), were classified as having Mild DR, characterized by early signs of damage to the retina. Moderate DR was observed in 40 patients (10.6%), indicating more advanced damage but without severe visual impairment. Severe DR was present in 30 patients (8%), marking a higher level of retinal damage and an increased risk of vision loss. Lastly, Proliferative DR, the most advanced form of the condition, involving neovascularization, was identified in 27 patients (7.1%). Table 3 includes measurements such as age, BMI, HbA1c, blood pressure, cholesterol levels, and the duration of diabetes. Notably, patients with DR tend to have higher values for BMI, HbA1c, fasting glucose, and duration of diabetes, which are all known risk factors for the development and progression of DR.

An analysis was conducted to examine the relationship between age and the presence of DR. The dataset was stratified into three age groups: <40 years, 40–59 years, and 60+ years. A Chi-Square test was performed to assess whether age significantly correlated with the presence of DR. The resulting *p*-value was 0.062, indicating that the correlation between age group and DR presence is not statistically significant at the conventional threshold of 0.05. However, the *p*-value suggests a trend toward higher DR prevalence in older age groups, though this relationship is not strong enough to reach statistical significance in this sample. This is supported by the Logistic Regression, which showed a non-significant result for this variable as well. A Chi-Square test for independence was also conducted to examine the relationship between gender and DR stages. The *p*-value > 0.05 indicated that the differences observed are not statistically significant. Smoking status does not appear to be a significant factor in the progression of DR, with an analysis that resulted in a *p*-value > 0.05.

**Figure 1:**
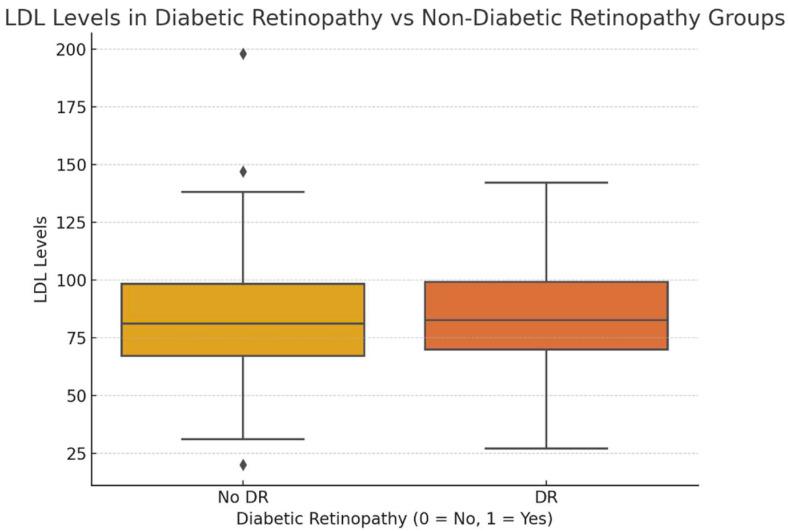
The boxplot visualizes LDL levels in individuals with and without diabetic retinopathy. The median LDL levels appear to be similar between both groups, and the distribution of LDL levels is also relatively similar. There are a few outliers in both groups, but, overall, there does not seem to be a significant difference in LDL levels between those with and without DR, which is consistent with the *t*-test results.

In the analysis comparing lipid profiles between diabetic patients with and without DR, no statistically significant differences were observed. For LDL levels, the t-statistic was 0.12 with a *p*-value of 0.90, indicating no meaningful difference between the two groups. Similarly, while HDL levels tended to be lower in the DR group, the t-statistic of −1.49 and the *p*-value of 0.14 suggest that this difference was not statistically significant. Finally, for triglycerides, the t-statistic of 0.99 and *p*-value of 0.32 confirmed no significant difference in triglyceride levels between the groups. These findings suggest that variations in lipid levels may not be critical factors in differentiating patients with or without DR in this dataset (Figure 1).

**Figure 2:**
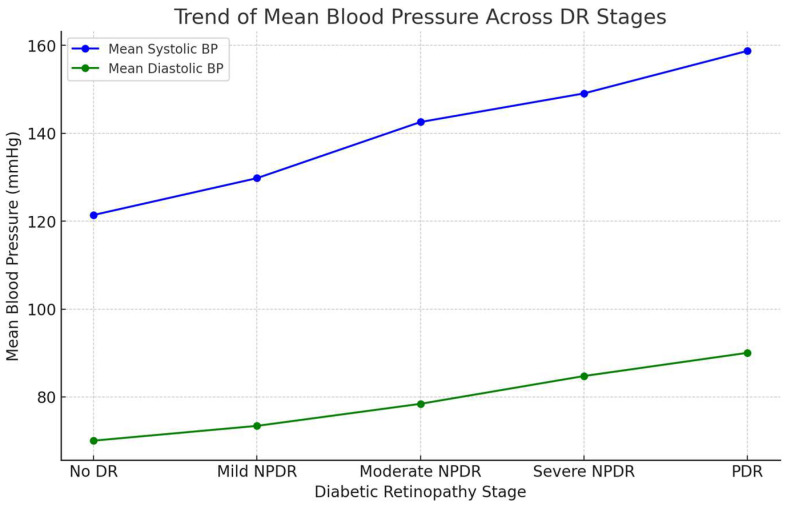
Trend in mean blood pressure across DR stages. This line graph illustrates the relationship between mean SBP and DBP and the progression of DR. As DR progresses, there is a clear upward trend in both systolic (blue line) and diastolic (green line) blood pressure, with the highest values being observed in patients with PDR.

Similarly, both systolic and diastolic blood pressure displayed moderate correlations with DR severity (Figure 2), with coefficients of 0.25 and 0.29, respectively. In the multivariate regression, these factors showed positive associations with more advanced stages of DR. Although the results were not statistically significant across all stages, the data suggest that elevated blood pressure is likely to increase the likelihood of more severe DR stages, such as Severe and Proliferative DR. These findings indicate that blood pressure is a moderate independent risk factor for DR progression, particularly in its association with advanced stages of the disease.

The analysis revealed that BMI has a moderate positive correlation with DR severity, with a correlation coefficient of 0.30. In the regression model, BMI demonstrated a positive coefficient in predicting higher DR stages. Although these coefficients were not statistically significant at all stages, the positive direction of the relationship suggests that a higher BMI may contribute to the progression of DR. This consistent association across stages supports the notion that BMI acts as a moderate independent risk factor for the development and severity of DR.

A Random Forest model was applied to predict the binary classification of No DR versus the presence of any DR stage. The model achieved an overall accuracy of 59.8%, correctly classifying over half of the patients. It performed particularly well in identifying patients with DR, showing a precision of 63% and a recall of 62% for this group, demonstrating its moderate ability to differentiate between patients who have DR and those who do not. The AUC (Area Under the Curve) for the Random Forest model in predicting DR versus No DR was 0.62. We used a 5-fold cross-validation to evaluate this model’s performance. The dataset was split into five parts, with the model trained on four parts and tested on the fifth. The Random Forest model achieved the following average results: an accuracy of 60.1% ± 3.2%, a precision of 61.3% ± 2.7%, a recall of 59.4% ± 3.5%, and an ROC-AUC of 0.62 ± 0.03

Among the most important features were BMI, which emerged as the strongest predictor in the model. Both DBP and SBP followed closely, indicating that blood pressure plays a critical role in the development of DR. HDL cholesterol also showed significant predictive value in the model despite not being statistically significant in basic tests. This suggests that HDL may contribute to the risk of DR when considered in combination with other factors. Additionally, total cholesterol, triglycerides, and LDL cholesterol played a meaningful role in the model’s predictions, though their importance was comparatively lower than that of BMI and blood pressure.

In addition to Random Forest, we applied XGBoost to evaluate its performance in predicting DR using cardiovascular risk factors. The model was evaluated using 5-fold cross-validation, with performance metrics being calculated across each fold. The average performance metrics for XGBoost showed an accuracy of 64.3% ± 2.7%, a precision of 63.5% ± 3.1%, a recall of 61.9% ± 2.9%, and an ROC-AUC of 0.68 ± 0.04. Compared to the Random Forest model, which achieved an ROC-AUC of 0.62, XGBoost demonstrated improved performance with an ROC-AUC of 0.68, indicating better discriminatory ability between patients. In addition, we also applied SVMs and Logistic Regression to evaluate their performance. For the SVM model, we observed an accuracy of 61.8% ± 3.0%, a precision of 60.7% ± 3.2%, a recall of 58.9% ± 3.4%, and an ROC-AUC of 0.65 ± 0.03. The SVM model showed moderate discriminatory ability but did not outperform XGBoost. However, it still performed better than Random Forest in terms of ROC-AUC. The Logistic Regression model achieved an accuracy of 59.7% ± 2.9%, precision of 58.5% ± 3.1%, recall of 57.1% ± 3.5%, and ROC-AUC of 0.60 ± 0.04. While Logistic Regression is a simpler model, its lower ROC-AUC compared to the other methods indicates that it may not capture the non-linear relationships present in the data as effectively. These findings suggest that XGBoost’s mechanism and SVM’s handling of complex patterns offer more accurate predictions in our case, making them better suited for predicting DR.

After tuning, the XGBoost model showed a notable improvement, with an ROC-AUC of 0.72 ± 0.03 compared to its pre-tuning score of 0.68. Similarly, the Random Forest model improved to an ROC-AUC of 0.65 ± 0.04. The SVM model also showed slight gains, with an ROC-AUC of 0.67 ± 0.03 after tuning the regularization parameter and kernel type. 

**Figure 3:**
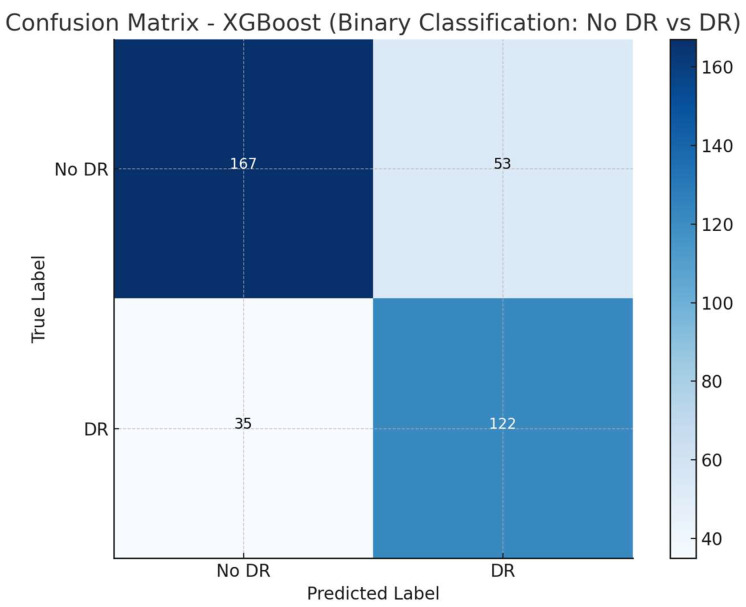
The confusion matrix for the XGBoost model predicting the presence of diabetic retinopathy. The model demonstrates moderate performance in distinguishing between patients with No DR and those with any stage of DR.

The confusion matrix (Figure 3) indicates that the model correctly classified 167 “No DR” cases and 122 “DR” cases while it incorrectly predicted 53 cases as “DR” when they were “No DR” (false positives) and 35 cases as “No DR” when they were actually “DR” (false negatives).

**Figure 4:**
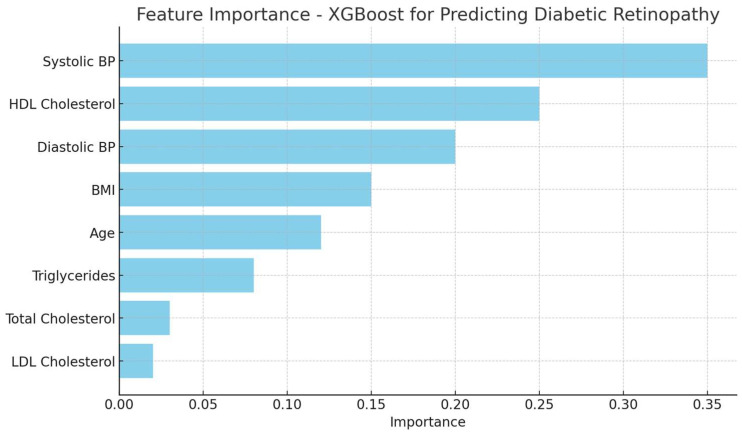
Feature importance in the XGBoost model for predicting diabetic retinopathy.

To address the need of enhancing the model’s interpretability, especially for clinical application, we conducted a feature importance analysis (Figure 4) which revealed that SBP, DBP, HDL cholesterol, and BMI were the most influential predictors in the model. 

Moreover, to further improve the transparency of the model, we applied SHAP (Shapley Additive exPlanations) values. The global feature importance analysis revealed that SBP had the highest average SHAP value of 0.35, indicating it was the strongest positive predictor of DR. HDL cholesterol, on the other hand, had an average SHAP value of −0.27, showing its protective effect, as higher HDL levels reduced the predicted risk of DR. BMI also played an important role, with an average SHAP value of 0.19, further reinforcing its contribution to DR risk.

Focusing on individual features, we observed that patients with SBP levels above 140 mmHg had SHAP values ranging from 0.30 to 0.50, indicating a significant increase in DR risk. For example, a patient with SBP of 150 mmHg had a SHAP value of 0.45, highlighting its strong influence on DR predictions. In contrast, patients with HDL levels below 40 mg/dL had consistently negative SHAP values of between −0.20 and −0.35, with a patient with a HDL level of 35 mg/dL showing a SHAP value of −0.32. Furthermore, the SHAP analysis showed a significant interaction between SBP and BMI. For patients with both high BP and obesity (BMI > 30), the combined SHAP values reached up to 0.55, indicating a compounded risk of DR. 

These enhancements not only improved the model’s clinical utility but also ensured that clinicians can more easily understand the contribution of each risk factor, making it possible to develop more personalized prevention and intervention strategies for patients at risk of DR.

The calibration plots showed that XGBoost was well calibrated, with predicted probabilities closely matching observed outcomes. Random Forest exhibited slight overconfidence at higher probabilities. The Hosmer–Lemeshow test results indicated no significant lack of fit for either model, with XGBoost yielding a *p*-value of 0.28 and Random Forest a *p*-value of 0.32.

**Figure 5:**
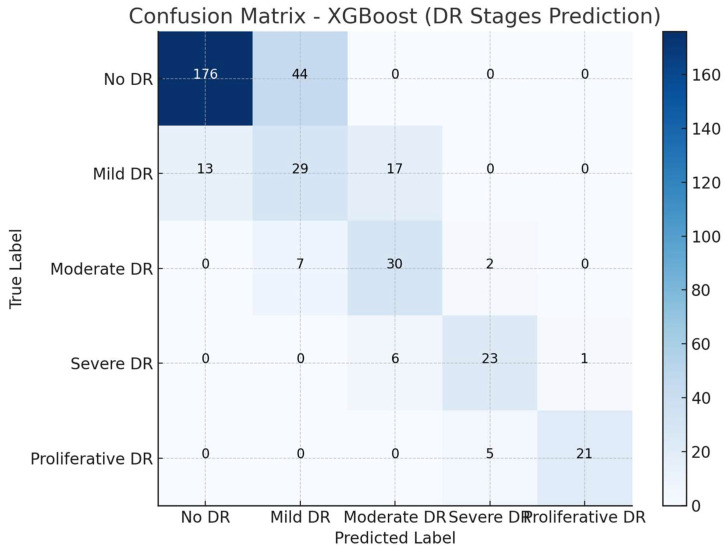
The confusion matrix for the XGBoost model predicting diabetic retinopathy stages.

A Random Forest model was also developed to predict the stages of DR based on the same cardiovascular risk factors (Figure 5). The model classified patients into four categories: No DR, Mild, Moderate, and Severe. The model demonstrated promising results, achieving an accuracy of 43.1% in predicting the DR stages. Most notably, the model showed strong performance in correctly identifying patients without DR, with a precision of 51% and a recall of 77% for the No DR class, indicating that it effectively differentiated between those without DR and those with more advanced stages. The weighted average precision and recall were also favorable, suggesting that the model was able to handle a range of DR stages. 

A XGBoost model was also developed to predict the stages of DR based on the same cardiovascular risk factors. This model demonstrated notable improvements, achieving an overall accuracy of 54.8% in predicting the stages of DR. The model performed particularly well in distinguishing patients without DR, with a precision of 63% and a recall of 85% for the No DR class, significantly improving the ability to correctly identify those without DR compared to the Random Forest model. This indicates that the XGBoost model was more adept at differentiating between patients with no signs of DR and those with more advanced stages. The weighted average precision and recall for all classes were 58% and 65%, respectively, suggesting that the model managed to handle various stages of DR with a better balance across the categories. Notably, the model also showed improved performance in predicting Mild and Moderate stages of DR, with a precision of 56% for the Mild category and 60% for Moderate cases, reflecting its potential for more accurate staging.

The XGBoost model showed a strong performance in classifying patients across the different stages of DR (Figure 5), though some misclassifications occurred, primarily between neighboring stages. For the No DR category, the model correctly classified 176 patients, while 44 patients were misclassified as having Mild DR. In the Mild DR category, the model correctly classified 29 patients, but 13 were misclassified as No DR and 17 were classified as having Moderate DR. For Moderate DR, the model correctly identified 30 patients, while seven were misclassified as Mild DR and two were misclassified as having Severe DR. In the Severe DR category, 23 patients were correctly classified, with six being misclassified as having Moderate DR. Lastly, for Proliferative DR, the model correctly classified 21 patients, but five were misclassified as having Severe DR. Misclassifications mostly occurred between adjacent DR stages, indicating that the model was effective in distinguishing between distinct stages, although there was some overlap between similar severity levels.

A Random Forest model was trained to predict the presence of PDR versus all other stages of DR. The model achieved an overall accuracy of 90.4%, correctly classifying a high proportion of cases. However, it primarily predicted non-PDR cases and struggled to identify actual cases of PDR. This outcome is likely due to the low prevalence of PDR in the dataset. These results suggest that, while the model effectively identifies non-PDR, its ability to detect PDR is limited by the low prevalence of this condition in the dataset.

## 4. Discussion

Risk factors for diabetic retinopathy encompass a wide range of physiological and lifestyle factors, including chronic hyperglycemia, hypertension, and dyslipidemia. Other significant risk factors include a longer duration of diabetes, poor glycemic control (high HbA1c levels), and obesity. Additionally, factors such as smoking, genetic predisposition, and coexisting cardiovascular conditions increase the likelihood of DR development [21]. Moreover, age and pregnancy can exacerbate DR risk, particularly in patients with existing diabetes [21,22].

Numerous research has looked into the risk factors for DR in various patient samples or groups. Previous research has demonstrated that the multiple factors influencing the onset of diabetes and DR, including the length of diabetes, blood glucose level, HbA1c, and high blood pressure, are what contribute to the multifaceted nature of DR [19,23]. Tsao et al. found that the administration of insulin and the length of diabetes were characteristics that could be used to identify individuals at high risk of DR by comparing many machine learning approaches [23]. Glucose levels is clearly not the sole factor that determines the incidence and course of DR, even though it is a crucial one [24]. An earlier investigation revealed a connection between glucose variability and the onset and course of diabetes; however, this association became less significant when hemoglobin A1c was taken into account [25]. 

Lately, a significant amount of research work has gone into creating machine learning algorithm-based predictive models for DR [21]. However, these models are not well suited for usage in settings with limited medical resources. Li et al. conducted a baseline analysis of sixty factors in their study, and the results indicated that insulin treatment also scored highly and that HbA1c was the most significant risk factor for DR; the risk increased with a HbA1c score of over 8% and insulin treatment being required. Age was also shown to be a protective factor for the onset and development of DR [15]. Research on the correlation between PDR and age has yielded conflicting results. The results are not always consistent, but some data indicate a possible increased risk of severe forms of the disease among younger people [26]. For instance, a review found that the risk of developing severe fibrovascular proliferation was considerably higher in those under 45. In a comparable direction, Klein et al. discovered that patients under 30 were more likely to develop PDR [27]. On the other hand, a prospective study found that the frequency of DR increased with age, while there was no statistically significant difference between the age groups [28]. Additionally, a national population-based cohort study revealed that people with DM who had had the disease for less than five years had a lower prevalence of DR and that regular screening had no effect on the detection rates of young patients (less than 45 years of age).

The rising incidence of obesity and insulin resistance has led to a large increase in the prevalence of diabetes mellitus and the diseases it is associated with. Despite being a significant risk factor for DR in people with type 2 diabetes, blood pressure and blood lipid levels may not always correlate. Concurrently, a significant body of research demonstrated that, in both non-diabetic and diabetic patients, central obesity, insulin resistance, and dyslipidemia are linked to retinal lesions [9]. Li et al. recruited 2305 patients and examined the relationship between obesity and any type of DR, diabetic macular edema (DME), and vision-threatening DR (VTDR). Their investigation yielded the following conclusions: only 29.6% of diabetic patients had normal weight, whereas 67.8% of diabetic patients were overweight or obese; second, the waist to height ratio was shown to be a substantial risk factor for VTDR, whereas obesity (BMI > 25.0 kg/m^2^) was shown to be a significant protective factor [29]. A prior meta-analysis assessing the relationship between central obesity and DR in the Chinese population found that, in contrast to the waist–hip ratio, abdominal obesity, as measured by waist circumference, is linked to an increased risk of DR. They did not, however, compare the outcomes across other ethnic groups. Furthermore, their investigation did not investigate if there is a similar correlation between abdominal obesity and DR in regard to varying severity [29,30]. 

Although there has been a considerable amount of clinical research performed on the relationship between DR and abdominal obesity, the findings have been inconsistent. Prior research found contradictory findings about the relationship between obesity and DR. The most common risk factor for DR was found to be obesity (BMI > 30 kg/m^2^) in cross-sectional research involving 501 persons with T1DM [31]. In Asian T2DM patients, a greater BMI seems to have an inhibitory impact on DR risk [32]. Prior cross-sectional investigations have likewise revealed an insignificant correlation between DR and BMI [33]. Several studies have shown that a higher BMI is associated with a higher risk of developing DR, particularly more severe forms. Some studies suggest that, for each unit increase in BMI, the risk of DR progression increases, although the exact mechanisms may vary among individuals.

Blood pressure plays a significant role in the progression of DR, with hypertension being a key modifiable risk factor [34]. Elevated blood pressure can exacerbate retinal blood vessel damage by increasing pressure on the fragile capillaries, leading to microaneurysms, hemorrhages, and worsening ischemia, all of which contribute to the progression of DR. The association between elevated blood pressure and diabetes mellitus was validated by research on epidemiology. Extensive, prospective studies such as UKPDS (The UK Prospective Diabetes Study) and ABCD (Appropriate Blood Pressure Control in Diabetes), including T2DM patients, indicate that strict blood pressure management can halt the onset and advancement of DR [35,36]. The study findings of Bulum et al. [4] imply that SBP and DBP serve as distinct risk factors for DR in T2DM patients. Even after accounting for the length of diabetes, gender, and HbA1c, there is a substantial correlation between the existence of DR and SBP in patients with type 2 diabetes regardless of whether their blood pressure is normal or elevated [37]. Regardless of the existence of cardiovascular and renal disease, SBP is also linked to both early and advanced DR [38]. In patients with T2DM, blood pressure variability, in addition to blood pressure degree, is a risk factor for DR [39]. While most research revealed a connection between DR and SBP, several studies found a relationship between DR and solely DBP [40]. 

Lipid levels and dyslipidemia have shown mixed results in their correlation with diabetes and the progression of DR. While some studies suggest that elevated LDL cholesterol and triglycerides, as well as low HDL cholesterol, may contribute to retinal damage, others have found the association to be less clear, indicating that the role of dyslipidemia in DR progression remains uncertain and may depend on additional risk factors [15,16,41,42]. In a comparable direction, there is an ongoing debate on the significance of lipoprotein(a) in the etiology of DR. The link between apolipoproteins and DR was the subject of a recent investigation; however, clinical practice has not yet made extensive use of these novel laboratory markers [43]. 

In our study, cardiovascular risk factors were evaluated for their association with DR. The most significant predictors of DR were found to be BMI, SBP and DBP. No significant relation was obtained between age, gender, and smoking status. 

### 4.1. Comparison of Different Models

One of the key strengths of the XGBoost model is its ability to handle complex, non-linear relationships in the data. DR is influenced by a variety of interrelated cardiovascular and metabolic factors, and XGBoost’s boosting approach allows for these interactions to be effectively captured and modeled. This flexibility makes it superior to linear models, such as Logistic Regression, which may oversimplify these relationships. XGBoost also offers excellent performance in handling imbalanced datasets. DR data, especially when subclassified, tends to be imbalanced, with a larger proportion of patients falling into the No DR or early-stage categories. XGBoost effectively mitigates this challenge through class weighting and boosting iterations, resulting in more accurate predictions for under-represented classes, such as Moderate and Severe DR, compared to models like Random Forest or SVMs, which may struggle with such imbalances. Another major advantage is XGBoost’s scalability and efficiency. Given that the dataset for this study contained hundreds of patients, the model was able to efficiently handle large volumes of data without significant computational strain thanks to its ability to parallelize the boosting process. This advantage is particularly important when dealing with clinical datasets that continue to grow in size. However, Random Forest offers higher interpretability and requires less fine-tuning, making it a more user-friendly model in cases where interpretability and ease of use are prioritized over predictive performance. Furthermore, tuning options for hyperparameters in XGBoost, such as learning rate, max depth, and regularization parameters, allowed for optimal model performance. The hyperparameter tuning process, including the adjustment of learning rate and class weights, enabled the model to generalize well across stages of DR without overfitting. In comparison, traditional models offer less flexibility in parameter optimization, which can limit their effectiveness.

Despite its strengths, the XGBoost model has some limitations. Its low interpretability can make it difficult for clinicians to understand the direct impact of individual risk factors on predictions, which is crucial in clinical settings. Tools like SHAP can enhance interpretability but add complexity compared to more transparent models like Logistic Regression. Additionally, XGBoost requires extensive tuning to achieve optimal performance, making it more challenging to implement than simpler models like Random Forest. Finally, while XGBoost excels in performance, it is computationally expensive and may require more resources and time, especially with large datasets, limiting its practicality in some clinical environments.

### 4.2. Clinical Application

One of the primary advantages of the XGBoost model is its ability to accurately predict the early onset of DR, particularly in patients with no visible signs of the disease. This predictive capability enables clinicians to intervene earlier, potentially delaying or preventing the progression of DR. For instance, patients identified as being at high risk of DR can be referred for more frequent retinal screening and aggressive management of modifiable risk factors, such as hypertension and dyslipidemia. Early intervention can help reduce the risk of progression to advanced stages of DR, where treatment options become more limited and complications more severe.

Furthermore, the model’s ability to distinguish between different stages of DR—No DR, Mild, Moderate, Severe, and Proliferative DR—provides valuable insights for stratifying patients according to their disease severity. This stratification allows clinicians to tailor treatment plans to individual patient needs. For example, patients predicted to have moderate or severe DR may benefit from more intensive therapeutic approaches, such as the early initiation of anti-VEGF therapy or laser treatment, to prevent further retinal damage and preserve vision.

Additionally, the XGBoost model incorporates cardiovascular risk factors that are routinely measured in clinical practice, making it highly adaptable for use in real-world healthcare settings. The simplicity of using widely available clinical data ensures that the model can be easily integrated into existing patient management workflows without the need for specialized or complex diagnostic procedures. This enhances its applicability in primary care settings, where early intervention is critical for preventing disease progression in diabetic patients. Another key aspect of the model’s utility is its ability to aid in decision-making regarding patient follow-up. By predicting the likelihood of progression to more advanced stages of DR, the model can inform decisions on how often patients should be monitored. 

Finally, the model’s potential extends beyond prediction to guiding treatment strategies. With accurate identification of patients at various stages of DR, clinicians can recommend interventions based on individual risk profiles. For instance, controlling blood pressure and optimizing lipid profiles in patients identified as being at risk of DR progression can prevent further retinal damage and improve long-term outcomes.

## 5. Limitations

While this study provides valuable insights into the relationship between clinical factors and the progression of DR, several limitations should be acknowledged. First, the sample size may have been insufficient for detecting statistically significant associations for certain analyses, particularly for more advanced stages like PDR, where the number of cases was relatively low. This imbalance can lead to suboptimal performance of predictive models, especially in accurately identifying minority classes. To address this, further studies can apply the Synthetic Minority Over-sampling Technique (SMOTE) to generate synthetic instances of the minority class, thus balancing the dataset. While SMOTE improves the model’s ability to detect advanced DR stages and helps mitigate bias toward the majority class, it may not fully compensate for the limited real-world representation of PDR cases.

Second, the dataset used was cross-sectional, capturing patient data at a single point in time. As a result, it is difficult to establish a clear cause-and-effect relationship between the clinical variables and DR progression. A longitudinal study following patients over time would provide more evidence for determining how factors like BMI, blood pressure, and lipid levels influence the development of DR. 

Improving the predictions of DR can be achieved through a combination of other advanced machine learning techniques and feature engineering. Incorporating domain-specific knowledge, addressing imbalanced data, and using regularization or dimensionality reduction techniques can also significantly enhance model performance. 

## 6. Conclusions

In this study, we evaluated cardiovascular risk factors as independent predictors of DR in patients with T2DM. Among the models tested, XGBoost demonstrated the best performance with an ROC-AUC of 0.72, identifying SBP, DBP, HDL cholesterol, and BMI as the most important predictors. The other models tested demonstrated also moderate predictive ability in distinguishing DR. Integrating these factors into clinical practice could enhance early detection and intervention strategies for DR by focusing on the management of blood pressure, cholesterol levels, and body weight, potentially delaying the onset or progression of DR in high-risk individuals. Given the increasing prevalence of T2DM and associated complications, these insights are vital for refining treatment approaches and focusing on modifiable risk factors to lower the impact of diabetic retinopathy. Further longitudinal research is necessary to validate these findings and improve predictive models for clinical use.

## Figures and Tables

**Table 1:** Diabetic retinopathy classification.

DR Classification	Description
No DR	No signs of diabetic retinopathy are present.
Mild DR	Early stages of DR, characterized by retinal microaneurysms
Moderate DR	More extensive damage to retinal blood vessels, but no severe vision loss at the time of study. - Microaneurysms; - Dot and blot hemorrhages; - Hard exsudates/cotton wool spots.
Severe DR	Significant retinal damage, leading to a higher risk of vision loss. - >20 intraretinal hemorrhages in each of the 4 quadrants; - Venous beading in 2 or more quadrants; - Prominent IRMS in at least one quadrant.
Proliferative DR	Advanced stage of DR, characterized by abnormal blood vessel growth in the retina. - Neovascularization; - vitreous/preretinal hemorrhage.

Abbreviations: DR-diabetic retinopathy; IRMA-intraretinal microvascular abnormality.

**Table 2:** Demographic characteristics and cardiovascular risk factors of all patients with type 2 diabetes included in the study.

Variable	Mean	SD (Standard Deviation)
Age (years)	55.15	15.81
BMI (kg/m^2^)	28.87	6.7
Systolic BP (mmHg)	130.53	17.41
Diastolic BP (mmHg)	83.88	9.93
Total Cholesterol (mg/dL)	162.83	29.26
HDL Cholesterol (mg/dL)	52	14.91
LDL Cholesterol (mg/dL)	77.75	26.74
Triglycerides (mg/dL)	119.41	67.96

Abbreviations: BP-blood pressure; BMI-body mass index; HDL-high-density lipoprotein; LDL-low-density lipoprotein.

**Table 3:** Comparative characteristics of patients with and without DR, highlighting key cardiovascular and metabolic parameters.

Parameter	Patients without DR (*n* = 220)	Patients with DR (*n* = 157)
Age (years)	54.2 ± 10.1	58.6 ± 9.8
BMI (kg/m^2^)	27.8 ± 3.5	30.1 ± 4.2
HbA1c (%)	7.5 ± 1.2	8.4 ± 1.4
Fasting Glucose (mg/dL)	140.6 ± 32.3	165.2 ± 40.1
Total Cholesterol (mg/dL)	180.1 ± 25.4	185.6 ± 28.3
LDL Cholesterol (mg/dL)	100.3 ± 19.5	102.8 ± 24.7
HDL Cholesterol (mg/dL)	48.7 ± 9.3	42.3 ± 8.8
Triglycerides (mg/dL)	160.2 ± 36.4	167.1 ± 42.7
Duration of Hypertension (years)	8.3 ± 5.7	11.2 ± 6.1
Systolic BP (mmHg)	130.5 ± 12.8	142.1 ± 15.2
Diastolic BP (mmHg)	82.1 ± 8.6	89.5 ± 10.3
Duration of Diabetes (years)	7.1 ± 4.5	13.6 ± 6.5
Smoking Status (% smokers)	35.2%	37.7%

## Data Availability

Data available upon request.

## References

[1] Wang W., Lo A.C.Y. (2018). Diabetic Retinopathy: Pathophysiology and Treatments. Int. J. Mol. Sci..

[2] Dănilă A.-I., Ghenciu L.A., Stoicescu E.R., Bolintineanu S.L., Iacob R., Săndesc M.-A., Faur A.C. (2024). Aldose Reductase as a Key Target in the Prevention and Treatment of Diabetic Retinopathy: A Comprehensive Review. Biomedicines.

[3] Munjral S., Maindarkar M., Ahluwalia P., Puvvula A., Jamthikar A., Jujaray T., Suri N., Paul S., Pathak R., Saba L. (2022). Cardiovascular Risk Stratification in Diabetic Retinopathy via Atherosclerotic Pathway in COVID-19/Non-COVID-19 Frameworks Using Artificial Intelligence Paradigm: A Narrative Review. Diagnostics.

[4] Bulum T., Tomić M., Vrabec R., Brkljačić N., Ljubić S. (2023). Systolic and Diastolic Blood Pressure Are Independent Risk Factors for Diabetic Retinopathy in Patients with Type 2 Diabetes. Biomedicines.

[5] Damle D., Karambelkar V. (2022). Study of Proliferative Diabetic Retinopathy and Its Correlation with Hypertension, Dyslipidaemia and Diabetic Nephropathy. Int. J. Health Sci..

[6] Rao H., Jalali J.A., Johnston T.P., Koulen P. (2021). Emerging Roles of Dyslipidemia and Hyperglycemia in Diabetic Retinopathy: Molecular Mechanisms and Clinical Perspectives. Front. Endocrinol..

[7] Singh A., Srinivasan A.K., Chakrapani L.N., Kalaiselvi P. (2019). LOX-1, the Common Therapeutic Target in Hypercholesterolemia: A New Perspective of Antiatherosclerotic Action of Aegeline. Oxid. Med. Cell. Longev..

[8] Zhu W., Wu Y., Meng Y.F., Xing Q., Tao J.J., Lu J. (2018). Association of Obesity and Risk of Diabetic Retinopathy in Diabetes Patients: A Meta-Analysis of Prospective Cohort Studies. Medicine.

[9] Mbata O., Abo El-Magd N.F., El-Remessy A.B. (2017). Obesity, Metabolic Syndrome and Diabetic Retinopathy: Beyond Hyperglycemia. World J. Diabetes.

[10] Cai X., Chen Y., Yang W., Gao X., Han X., Ji L. (2018). The Association of Smoking and Risk of Diabetic Retinopathy in Patients with Type 1 and Type 2 Diabetes: A Meta-Analysis. Endocrine.

[11] Li J., Liu S., Cao G., Sun Y., Chen W., Dong F., Xu J., Zhang C., Zhang W. (2018). Nicotine Induces Endothelial Dysfunction and Promotes Atherosclerosis via GTPCH1. J. Cell. Mol. Med..

[12] Al Saad M., Shehadeh A., Hizzani A., Alzibdeh A., Alsubhi A.A., Hamdan D., Alkubati E., Meqbil J., Hamadneh L., Ababneh O. (2023). Effects of Smoking on Outcomes of Anti-Vascular Endothelial Growth Factor Therapy in Patients with Diabetic Macular Edema: A Retrospective Case-Control Study. Middle East Afr. J. Ophthalmol..

[13] King P., Peacock I., Donnelly R. (1999). The UK Prospective Diabetes Study (UKPDS): Clinical and Therapeutic Implications for Type 2 Diabetes. Br. J. Clin. Pharmacol..

[14] Buse J.B., Bigger J.T., Byington R.P., Cooper L.S., Cushman W.C., Friedewald W.T., Genuth S., Gerstein H.C., Ginsberg H.N., ACCORD Study Group (2007). Action to Control Cardiovascular Risk in Diabetes (ACCORD) Trial: Design and Methods. Am. J. Cardiol..

[15] Li W., Song Y., Chen K., Ying J., Zheng Z., Qiao S., Yang M., Zhang M., Zhang Y. (2021). Predictive model and risk analysis for diabetic retinopathy using machine learning: A retrospective cohort study in China. BMJ Open.

[16] Zhao Y., Li X., Li S., Dong M., Yu H., Zhang M., Chen W., Li P., Yu Q., Liu X. (2022). Using machine learning techniques to develop risk prediction models for the risk of incident diabetic retinopathy among patients with type 2 diabetes mellitus: A cohort study. Front. Endocrinol..

[17] Ghenciu L.A., Hațegan O.A., Stoicescu E.R., Iacob R., Șișu A.M. (2024). Emerging Therapeutic Approaches and Genetic Insights in Stargardt Disease: A Comprehensive Review. Int. J. Mol. Sci..

[18] Ghenciu L.A., Șișu A.M., Stoicescu E.R., Dănilă A.-I., Iacob R., Săndesc M.-A., Hațegan O.A. (2024). Thyroid Eye Disease and Glaucoma: A Cross-Sectional Study Comparing Clinical Characteristics and Disease Severity. Medicina.

[19] Yang Q.H., Zhang Y., Zhang X.M., Li X.R. (2019). Prevalence of diabetic retinopathy, proliferative diabetic retinopathy and non-proliferative diabetic retinopathy in Asian T2DM patients: A systematic review and meta-analysis. Int. J. Ophthalmol..

[20] Collins G.S., Reitsma J.B., Altman D.G., Moons K.G. (2015). Transparent Reporting of a Multivariable Prediction Model for Individual Prognosis or Diagnosis (TRIPOD): The TRIPOD Statement. BMC Med..

[21] Yang Y., Tan J., He Y., Huang H., Wang T., Gong J., Liu Y., Zhang Q., Xu X. (2023). Predictive Model for Diabetic Retinopathy under Limited Medical Resources: A Multicenter Diagnostic Study. Front. Endocrinol..

[22] Chung Y.C., Xu T., Tung T.H., Chen M., Chen P.E. (2022). Early screening for diabetic retinopathy in newly diagnosed type 2 diabetes and its effectiveness in terms of morbidity and clinical treatment: A nationwide population-based cohort. Front. Public Health.

[23] Tsao H.Y., Chan P.Y., Su E.C.Y. (2018). Predicting diabetic retinopathy and identifying interpretable biomedical features using machine learning algorithms. BMC Bioinform..

[24] Song P., Yu J., Chan K.Y., Theodoratou E., Rudan I. (2018). Prevalence, risk factors and burden of diabetic retinopathy in China: A systematic review and meta-analysis. J. Glob. Health.

[25] Wakasugi S., Mita T., Katakami N., Okada Y., Yoshii H., Osonoi T., Nishida K., Shiraiwa T., Torimoto K., Kurozumi A. (2021). Associations between continuous glucose monitoring-derived metrics and diabetic retinopathy and albuminuria in patients with type 2 diabetes. BMJ Open Diabetes Res. Care.

[26] Wu Y.B., Wang C.G., Xu L.X., Chen C., Zhou X.B., Su G.F. (2020). Analysis of risk factors for progressive fibrovascular proliferation in proliferative diabetic retinopathy. Int. Ophthalmol..

[27] Klein R., Klein B.E., Moss S.E., Davis M.D., DeMets D.L. (1984). The Wisconsin epidemiologic study of diabetic retinopathy. III. Prevalence and risk of diabetic retinopathy when age at diagnosis is 30 or more years. Arch. Ophthalmol..

[28] Anwar S.B., Asif N., Naqvi S.A., Malik S. (2019). Evaluation of multiple risk factors involved in the development of diabetic retinopathy. Pak. J. Med. Sci..

[29] Li W., Gong X., Wang W., Xiong K., Meng J., Li Y., Wang L., Liang X., Jin L., Huang W. (2022). Association of different kinds of obesity with diabetic retinopathy in patients with type 2 diabetes. BMJ Open.

[30] Rosu L.M., Prodan-Bărbulescu C., Maghiari A.L., Bernad E.S., Bernad R.L., Iacob R., Stoicescu E.R., Borozan F., Ghenciu L.A. (2024). Current Trends in Diagnosis and Treatment Approach of Diabetic Retinopathy during Pregnancy: A Narrative Review. Diagnostics.

[31] Price S.A., Gorelik A., Fourlanos S., Colman P.G., Wentworth J.M. (2014). Obesity is Associated with Retinopathy and Macrovascular Disease in Type 1 Diabetes. Obes. Res. Clin. Pract..

[32] Man R.E., Sabanayagam C., Chiang P.P., Li L.J., Noonan J.E., Wang J.J., Wong T.Y., Cheung G.C., Tan G.S., Lamoureux E.L. (2016). Differential Association of Generalized and Abdominal Obesity with Diabetic Retinopathy in Asian Patients with Type 2 Diabetes. JAMA Ophthalmol..

[33] Wang J., Chen H., Zhang H., Yang F., Chen R.P., Li Y.B., Yang C., Lin S.D., Chen L.S., Liang G.X. (2014). The Performance of a Diabetic Retinopathy Risk Score for Screening for Diabetic Retinopathy in Chinese Overweight/Obese Patients with Type 2 Diabetes Mellitus. Ann. Med..

[34] Schreur V., Ng H., Nijpels G., Stefánsson E., Tack C.J., Klevering B.J., de Jong E.K., Hoyng C.B., Keunen J.E., van der Heijden A.A. (2021). Validation of a model for the prediction of retinopathy in persons with type 1 diabetes. Br. J. Ophthalmol..

[35] Matthews D.R., Stratton I.M., Aldington S., Holman R.R., Kohner E.M., UK Prospective Diabetes Study Group (2004). Risks of progression of retinopathy and vision loss related to tight blood pressure control in type 2 diabetes mellitus (UKPDS 69). Arch. Ophthalmol..

[36] Schrier R.W., Estacio R.O., Mehler P.S., Hiattet W.R. (2007). Appropriate blood pressure control in hypertensive and normotensive type 2 diabetes mellitus: A summary of the ABCD trial. Nat. Clin. Pract. Nephrol..

[37] Li Y.T., Wang Y., Hu X.Y., Chen J.H., Li Y.Y., Zhong Q.Y., Cheng H., Mohammed B.H., Liang X.L., Hernandez J. (2021). Association between Systolic Blood Pressure and Diabetic Retinopathy in Both Hypertensive and Normotensive Patients with Type 2 Diabetes: Risk Factors and Healthcare Implications. Healthcare.

[38] Liu L., Quang N.D., Banu R., Kumar H., Tham Y.-C., Cheng C.-Y., Wong T.Y., Sabanayagam C. (2020). Hypertension, blood pressure control and diabetic retinopathy in a large population-based study. PLoS ONE.

[39] Lou Q., Chen X., Wang K., Liu H., Zhang Z., Lee Y. (2022). The Impact of Systolic Blood Pressure, Pulse Pressure, and Their Variability on Diabetes Retinopathy among Patients with Type 2 Diabetes. J. Diabetes Res..

[40] Rajalakshmi R., Amutha A., Ranjani H., Ali M.K., Unnikrishnan R., Anjana R.M., Narayan K.M.V., Mohan V. (2014). Prevalence and risk factors for diabetic retinopathy in Asian Indians with young onset type 1 and type 2 diabetes. J. Diabetes Complicat..

[41] Ezhilvendhan K., Sathiyamoorthy A., Prakash B.J., Bhava B.S., Shenoy A. (2021). Association of Dyslipidemia with Diabetic Retinopathy in Type 2 Diabetes Mellitus Patients: A Hospital-Based Study. J. Pharm. Bioallied Sci..

[42] Dumitrescu A., Vitcu G.M., Stoica S., Susa S.R., Stoicescu E.R. (2024). Cardiovascular Risk Factors in Socioeconomically Disadvantaged Populations in a Suburb of the Largest City in Western Romania. Biomedicines.

[43] Zhang X., Nie Y., Gong Z., Zhu M., Qiu B., Wang Q. (2022). Plasma apolipoproteins predicting the occurrence and severity of diabetic retinopathy in patients with type 2 diabetes mellitus. Front. Endocrinol..

